# Pollinator probing preference and switching mode-mediated self-interference within a monoecious plant significantly reduced reproductive fitness

**DOI:** 10.3389/fpls.2023.1243764

**Published:** 2023-10-10

**Authors:** Bi-Xian Wu, Li-Na Ma, Nan Xia, Hao Wang, Guo-Xing Cao

**Affiliations:** ^1^ Key Laboratory of National Forestry and Grassland Administration on Forest Resources Conservation and Ecological Safety in the Upper Reaches of the Yangtze River, College of Forestry, Sichuan Agricultural University, Chengdu, China; ^2^ Langzhong Natural Resources and Planning Bureau, Langzhong, China; ^3^ Langzhong Agricultural Bureau, Langzhong, China; ^4^ College of Life Science, Yan’an University, Yan’an, China

**Keywords:** *Akebia trifoliata*, monoecy, pollen limitation, pollinator behavior, selfing, self-compatibility, self-interference, reproductive fitness

## Abstract

**Introduction:**

Monoecy is usually interpreted as an important evolutionary route of the plant sexual system from hermaphroditism to dioecy. This floral mechanism can effectively reduce self-interference during the reproductive process, and the services provided by pollinators may play an essential role in monoecious species; however, relevant research is still lacking. Thus, we aimed to determine whether monoecious plants could effectively avoid self-interference and promote the evolution of monoecy under the service of pollinators.

**Methods:**

Here, we successfully performed manipulation experiments to test self-compatibility, pollinator behavior, and self-interference between male and female functions in *Akebia trifoliata*, a typical monoecious species.

**Results:**

We demonstrated that experimental self-pollination did not yield any fruit, and supplemental pollination significantly increased fruit set and fruit weight compared to natural pollination, suggesting that this species is completely self-incompatible and experiences strong pollen limitation. Simultaneous self- and cross-pollination and self-pollination prior to cross-pollination significantly reduced reproductive fitness, but self-pollination after cross-pollination did not, indicating self-interference in this plant. Moreover, both male flower probing preference and switching modes within inflorescences by pollinators successfully reinforced self-interference and were also responsible for decreasing reproductive fitness in *A*. *trifoliata*.

**Discussion:**

In summary, pollinator-mediated self-interference significantly reduced selfing, providing potential dynamics for the maintenance and evolution of monoecy.

## 1 Introduction

Self-interference occurs when the male and female functions of a plant interfere with one another, either physically or biochemically, potentially reducing both maternal and paternal fitness (Cesaro et al., 2004; Li et al., 2013). During the reproductive process of flowering plants, flowers often receive self-pollen from the same flower and/or other flowers within the same plant, resulting in self-pollination (Aizen and Harder, 2007). Self-pollination may interfere with cross-pollination performance, potentially causing self-interference (Barrett, 2002a), particularly in self-incompatible species. Plants have evolved many floral mechanisms to reduce self-interference (Lloyd and Yates, 1982; Barrett, 2002a), such as the floral traits of herkogamy, dichogamy, and monoecy (Bawa and Beach, 1981; Bertin, 1993).

Many studies have tested within-flower self-interference in herkogamous and/or dichogamous species (Cesaro et al., 2004; Routley and Husband, 2006; Dai and Galloway, 2011; Navarro et al., 2012; Li et al., 2013; Guo et al., 2014; Xiao et al., 2017); however, few studies have investigated self-interference and discussed its role in the reproduction of monoecious plants (Kawagoe and Suzuki, 2005; Torres and Puntieri, 2013). Monoecy refers to a stable sexual system in which plants bear both unisexual female and male flowers on the same individual. It is thought to be an important middle stage in the evolution of the angiosperm sexual system from hermaphroditism to dioecy (a dimorphic sexual system in which populations are composed of male and female plants) (Barrett, 2002b). Although the most obvious function of unisexual flowers in monoecious species is to reduce self-interference by avoiding geitonogamy (Darwin, 1876; Lloyd and Webb, 1986; Webb and Lloyd, 1986; Harder et al., 2000), the magnitude of self-interference varies during pollinator-mediated pollination because it is governed by the number of simultaneously presented female and male flowers in monoecious species. In addition, a lower magnitude of self-interference can significantly prevent selfing and further promote the evolution of separating sexes from both sexes (Charlesworth and Charlesworth, 1978; Lloyd, 1982; Charlesworth, 1999). Unfortunately, there has been no comprehensive research investigating the mechanisms of monoecious species to avoid self-interference and selfing.

The services provided by pollinators may play an important role in preventing self-interference in monoecious plants. Like most flowering plants, most monoecious species rely on pollinators to transport pollen grains to ovules for reproduction, but few or ineffective pollinator visits could reduce the quantity or quality of fitness (Knight et al., 2005; Aizen and Harder, 2007). For example, in monoecious species, male fitness may be reduced because of pollen discounting in self-incompatible species, i.e., a decrease in the availability of pollen for outcrossing (Harder and Wilson, 1998). When self-pollen occupies the surface area of the stigma, female fitness may be reduced because the deposition of self-pollen may cause stigmatic or stylar clogging and usurp or disabled ovules, which are then unavailable for cross-fertilization, preventing subsequent fruit and/or seed development in self-incompatible species (Husband and Schemske, 1996; Barrett, 2002a; Kawagoe and Suzuki, 2005; Torres and Puntieri, 2013). In addition, pollinator probing preference and switching mode (the variation pattern of pollinator visits between female and male flowers) within inflorescences will significantly influence the quality of pollen received by the stigma and potentially cause self-interference in monoecious plants. For instance, if pollinators visit male flowers followed by female flowers, self-interference is unavoidable, resulting in pollen discounting and stigma clogging, especially in self-incompatible species. In contrast, if a pollinator visits female flowers followed by male flowers, it could help the plant gain higher female and male fitness by avoiding self-interference. Thus, we assumed that pollinator services are critical to the occurrence of self-interference in monoecious species, and it is important to explore the relationship between pollinators and self-interference to understand reproduction in monoecious species. However, few studies have directly explored the potential effects of pollinator behavior on self-interference and reproductive fitness.

*Akebia* species are typical monoecious woody vines in the Yangtze River basin of China, occurring widely in brushwood between 250 and 2,000 m (Ekiert et al., 2021), providing sampling convenience to explore the effects of self-interference and pollinator services on reproductive success. In addition, as a traditional medicinal plant, *Akebia* species have been used in Chinese herbalism for at least 2,000 years. Among these, *A*. *quinata* (Houtt) Decne and *A*. *trifoliata* (Thunb) Koidz are currently recorded as medicinal plants in the Chinese and Japanese Pharmacopoeia (Kawasaki and Higuchi, 1976; Ekiert et al., 2021). *A*. *trifoliata* is rich in nutrients and has health benefits and other newly discovered fruit properties during the primary stages of domestication, making it worthy of exploitation as a new high-value crop (Li et al., 2010; Iketani, 2016; Ekiert et al., 2021). Thus, from the perspective of crop management, it is also important to understand that fruit production in *A*. *trifoliata* is limited by self-interference. In the present study, we assumed that *A*. *trifoliata* is self-incompatible, similar to other plants in the genus, and that reproductive fitness is influenced by pollen limitation through pollinator probing. Moreover, because female flowers are 3.5 times larger than male flower and exhibit a greater advertising effect on pollinators, the pollinator may exhibit a significant female probing preference, which could successfully enhance reproductive fitness in *A. trifoliata* by preventing self-interference. To test this hypothesis, we first conducted artificial pollination treatments to assess the influence of self-pollination on outcrossed fruit production. We then investigated the effects of pollinator probing preference and switching modes within an inflorescence on self-interference using field observations. In addition, we experimentally manipulated flower size using a trimming treatment to explore the impact of flower size on pollinator probing preference, self-interference, and reproductive success.

## 2 Materials and methods

### 2.1 Study species and populations

**Figure 1:**
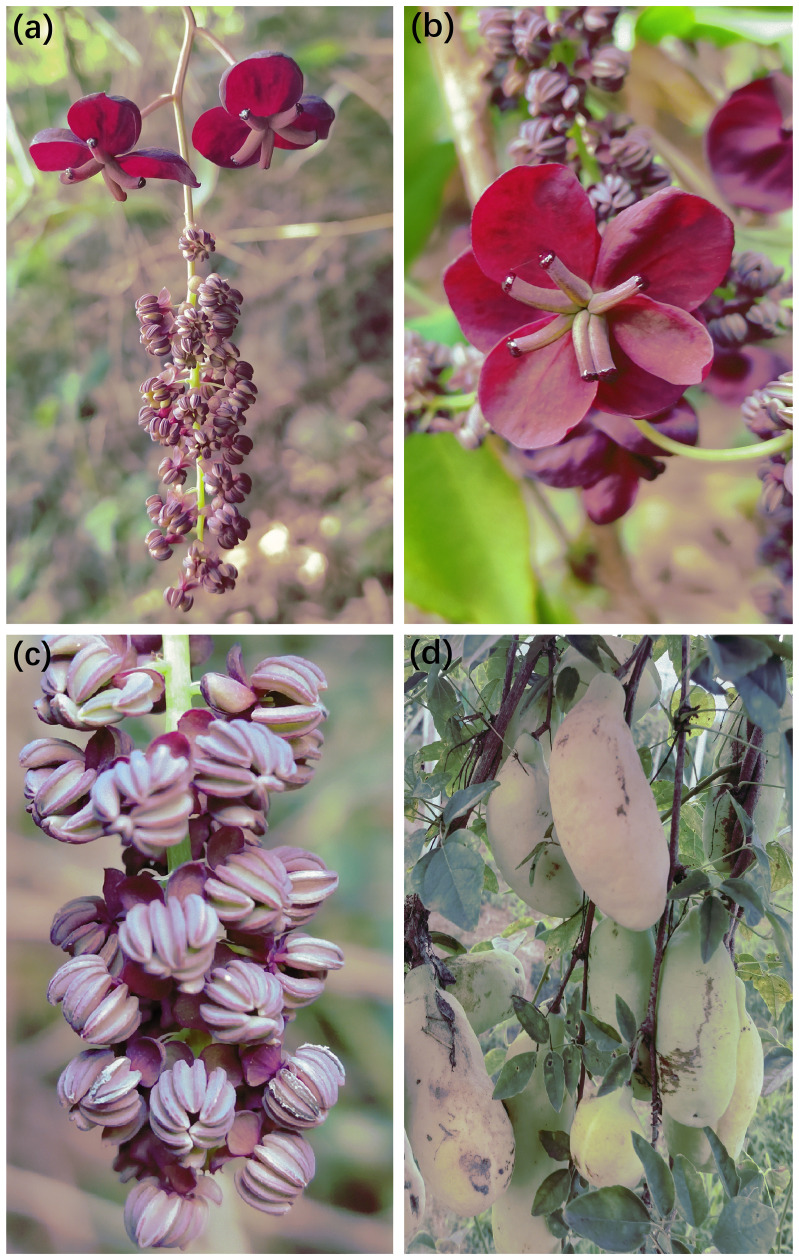
Photographs of inflorescence morphology **(A)**, female flower **(B)**, male flower **(C)** and fruit **(D)** in *Akebia trifoliata*. The photographs of the LY population were taken by Liu Ling.

*Akebia trifoliata* (Lardizabalaceae) is widespread in mountainous areas of China (Li et al., 2010). The major pollinator is the honeybee. Individual plants can produce hundreds of inflorescences and simultaneously open many female and male flowers (**Figure 1A**). Flowers are nectarless but have a sweet aroma (**Figure 1B**). Within an inflorescence, male flowers commonly have six deep-purple stamens and pollen is the only reward for pollinators (**Figure 1C**). Female flowers were larger than male flowers (**Figure 1A**), petals were absent, and three to 12 divergent purple carpels appeared to function in pollinator attraction (**Figure 1B**). Each carpel bears a stigma and subsequently develops a fruit from late August to early September (**Figures 1B-D**). In addition, female flowers are proximal and open several days earlier than distal male flowers (protogynous), but overlapping periods occur within inflorescences; therefore, self-interference may occur in *A. trifoliata*.

The experiments were conducted in Sichuan Province, Southwest China, including cultivated and wild populations. The cultivated population was located at the Chongzhou (CZ) base of Sichuan Agricultural University (30°33′36″N, 103°39′26″E; 513 m a.s.l.). All individuals were transplanted from Shimian County, Sichuan Province, China, and planted in rows spaced 2 m apart in the early spring of 2018. The orchard covered approximately 4,000 m^2^. This region experiences a subtropical humid monsoon climate with an average annual temperature of 17.5°C and an average annual precipitations of 1,122.4 mm (meteorological data were obtained from the Chongzhou Meteorological Bureau). The soil type was yellow soil, viscous, and acidic. Non-irrigation but organic and compound fertilizers were applied to the common garden. The wild population is located on Laoya Mountain (LY), Wolong National Natural Reserve (31°5′19″N, 103°17′56″E; 1680 m a.s.l.). This region experiences a temperate monsoon climate with an average annual temperature of 10.0°C and an average annual precipitation of 883.7 mm (meteorological data were obtained from the Wenchuan Meteorological Bureau). The soil type was yellow-brown soil, viscous, and subacidic. The flowering plants in this population were sparsely distributed, with interplant distances ranging from 15 to 30 m. The flowering period of *A. trifoliata* was from mid-February to late March in the CZ cultivated population, and from late March to late April in the LY wild population. The longevity of individual male flowers (the time between anthesis and senescence of the calyx) was 4.57 ± 0.19 and 4.93 ± 0.31 days for the two populations, respectively (both *n* = 30). The longevity of individual female flowers was 17.30 ± 0.55 and 23.57 ± 0.90 days for the two populations, respectively (both *n* = 30).

### 2.2 Self-compatibility and pollen limitation

In the CZ cultivated and LY wild populations, 30 plants were randomly tagged before flowering in 2019 and 2021, respectively. Flowers or fruits in different positions within plants may be affected by differences in resource-allocation strategies. Thus, we tagged three female flowers (at the basal, middle, and distal positions) on each branch at the budding stage, and five branches were selected per plant. Five branches on each tagged plant were randomly assigned to the following pollination treatments: (1) Natural pollination: flower buds were left intact and open to pollinators and wind during flowering. (2) Bagging: Female flower buds were wrapped in sulfuric acid paper bags (to prevent both wind and insect pollination) to test whether apomixis occurred in *A*. *trifoliata*. The bags were removed after flower wilting. (3) Netting: female flower buds were wrapped in nylon mesh nets (1-mm mesh) to prevent insect access but allow wind pollination. The nets were removed after flower wilting. (4) Self-pollination: female flower buds were covered with nylon mesh nets. When the stigmas became receptive, we manually pollinated them with pollen from the male flowers of the same inflorescence. The nets were removed after flower wilting. (5) Supplemental pollination: Receptive stigmas were hand-pollinated with cross pollen from other plants and exposed to natural pollination. Pollen is collected from at least two donor individuals, and stored in a centrifuge tube, the distance between the pollen recipient and pollen donor was always at least 10 m. Sufficient pollen was applied to receptive stigmas within 2 h using a thin paint brush. In addition, to test whether experimental plants could reallocate resources away from natural inflorescences to supplemental inflorescences within plants, another 30 neighboring unmanipulated plants were randomly tagged before flowering in the CZ-cultivated population in 2019. We tagged one branch on each plant, and three female flowers (from the basal, middle, and distal positions) were allowed to undergo natural pollination until the flowers withered, as an external control treatment.

Fruits were harvested at maturation in early September, oven-dried at 70°C for 48 h to a constant weight and weighed to the nearest 0.01 mg (Zhang et al., 2014). The fruit set per plant was estimated as the number of flowers producing at least one mature fruit divided by the total number of flowers. The fruit dry biomass was estimated as the total dry weight of all mature fruits divided by the number of flowers that set the fruit. The pollen limitation index was calculated as PL = 1 − (*P_o_*/*P_s_*), where *P_o_* is the fruit set resulting from natural pollination and *P_s_* is the result of supplemented cross-pollination (Larson and Barrett, 2000). The calculations for the two populations were performed separately.

### 2.3 Flower traits and pollinator probing behavior

To determine the number of flowers per inflorescence, 32 plants were randomly selected and used to estimate the number of flowers in CZ and LY populations. Similarly, to effectively eliminate the effect of inflorescence position on flower traits, we tagged three inflorescences (from the basal, middle, and distal positions) per plant to count female and male flowers separately until the end of anthesis. In addition, to estimate the difference in flower size and flower dry biomass between female and male flowers, we selected thirty-three plants per population in CZ and LY. In each plant, three inflorescences were randomly selected to measure sepal diameter using digital calipers, and each inflorescence contained two female and male flowers. After the measurement, all flowers were collected to determine the difference in dry biomass between the sexes. All flowers were oven-dried at 70°C for 48 h to a constant weight in the laboratory and then weighed to the nearest 0.01 mg (Zhang et al., 2014). We adopted the average weight (the total female/male dry weight divided by the total female/male number) to indicate the dry biomass per female or male flower, because this method can effectively avoid the position effect on flower dry biomass.

To examine the probing behavior of honeybees within inflorescences on *A. trifoliata*, including the probing bias (the first foraging choice between male and female flowers), the per-flower visitation rate, and the proportion of pollinators switching probing between male and female flowers, we observed pollinator behavior at peak flowering in the CZ cultivated population in 2019. A total of 124 inflorescences were randomly selected for this experiment on consecutive sunny and windless days. The inflorescences selected were from the basal, middle, and distal positions, and this sampling strategy effectively avoided the interference of position on pollinator visitation and self-interference. Before the observation, the number of female and male flowers per inflorescence was recorded, and then the number of flowers visited by pollinators, pollinator probing sequences, and switching trajectories between female or male flowers within inflorescences were recorded until the pollinators left the target inflorescence. The pollinator switching modes included the following: M or F indicates that the pollinator probed only male or female flowers, respectively; MM or FF indicates the switching of probing within male or female flowers, respectively; MF indicates that the pollinator probed male flowers followed by female flowers, and FM was the opposite of MF. The observation time started at 09:00 and ended at 16:00 each day. Visitation rate was calculated as the number of visits per flower per day. In addition, to explore whether flower size significantly influences pollinator probing frequency, selection preference, and switching modes, 124 inflorescences were randomly selected for observation experiments. During this experimental process, all the female flower sepals were trimmed with scissors to keep the female and male flowers the same size.

### 2.4 Self-interference

Exploring the existence of self-interference in *A*. *trifoliata.* A total of 66 flowering plants were randomly selected before flowering at the CZ cultivation base of Sichuan Agricultural University in 2021, and the large cultivation base was split into two populations, CZ1 and CZ2. Three female flowers were tagged and covered with nylon mesh nets (from the basal, middle, and distal positions) per branch at the budding stage, and five branches were selected per plant. The five branches were randomly assigned to the following five pollination treatments: (1) Cross-pollination (CP-CP): Cross-pollination followed by cross-pollination 24 h later. (2) Self-pollination (SP-SP): Self-pollination followed by self-pollination 24 h later. (3) Cross-pollination followed by self-pollination 24 h later (CP-SP). (4) Self-pollination followed by cross-pollination 24 h later (SP-CP). (5) Simultaneous cross-pollination and self-pollination (MP-MP): A mixture of cross-pollen and self-pollen was applied to receptive stigmas and again 24 h later. All experimental manipulations and time intervals were performed as described by Kawagoe and Suzuki (2005). Pollen is collected from at least two donor individuals, and stored in a centrifuge tube, the distance between the pollen recipient and pollen donor was always at least 10 m. Sufficient pollen was applied to receptive stigmas within 2 h using a thin paint brush, and the ratio of cross-pollen and self-pollen was 1:1. All female flowers were wrapped with nylon nets, which were removed after flower wilting. Fruits were harvested at maturation in early September, oven-dried at 70°C for 48 h to a constant weight and weighed to the nearest 0.01 mg (Zhang et al., 2014). Fruit set per flower per plant was estimated as the number of flowers producing at least one mature fruit divided by the number of flowers. In addition, one female flower may have several divergent fruits because the female flowers have three to 12 divergent carpels, and each carpel has the potential to develop into fruit. Therefore, fruit set per carpel per flower was estimated as the number of carpels producing mature fruit divided by the number of carpels.

### 2.5 Statistical analyses

All statistical analyses were conducted using R (version 4.1.1; R Core Team, 2018). Generalized linear mixed models (GLMMs) were fitted using the package *lme4* (Bates et al., 2015). The *car* package was also used if categorical variables were treated as factors in the GLMM or generalized linear model (GLM) (Fox and Weisberg, 2011). Pairwise comparisons of the estimated marginal means were performed using the *emmeans* package (Lenth, 2018).

First, to test the effects of sex, population, and their interaction on flower traits (including number of flowers, flower size, and dry biomass), we treated sex and population as categorical and fixed factors in the GLM model, and binomial distribution (logit link) and normal distribution (identity-link) were applied to flower number and flower size/dry biomass, respectively. To detect the differences in flower traits between sexes within the population, pairwise comparisons were performed using the *emmeans* package. Second, the Shapiro−Wilk normality test results indicated that the data on fruit set, fruit dry biomass, pollinator selection preference, and visitation rate per flower did not follow a normal distribution. Therefore, we also used GLM to detect the effects of pollination treatment, population, and their interactions on fruit set and fruit dry biomass. Binomial distribution (logit-link) was applied to fruit set data, and normal distribution (identity-link) was applied to fruit dry biomass data. In these analyses, pollination treatment and population were treated as fixed and categorical factors, respectively. In addition, because we used an hour as the unit of time, and the per-flower visitation rate was indicated by the number of visits divided by the total number of flowers, we tested the effects of sex, population, and their interaction on the pollinator visitation rate per flower using a GLM model with a binomial distribution and logit-link function. Moreover, the number of flowers can significantly influence pollinator selection preferences; thus, GLMM was used to detect the effects of sex, population and their interaction on pollinator selection preferences. We treated the flower number as a random factor, and Poisson distribution (log-link) was used in this analysis. Third, we used a GLM to explore whether the trimming treatment influenced the pollinator probing sequence and switching between female and male flowers by comparison with the natural treatment. We used the marginal effect of the means analysis methods to detect whether the different switching modes significantly differed between the natural and trimming treatments. A binomial distribution (logit link) was applied in the analysis. Finally, for each GLM and GLMM, we checked for overdispersion using the equation “deviance (fit.reduced)/df.residual (fit.reduced),” and if overdispersion was detected, we adopted the “quasi-Poisson” or “quasi-Binomial” distribution family to replace the “Poisson” or “Binomial” family. To compare fruit set, fruit dry biomass, pollinator selection preference and visitation rate per flower between sexes, populations, and pollination treatments, we performed pairwise comparisons using the *emmeans* package in R with the estimated marginal means (Lenth, 2018).

## 3 Results

### 3.1 Flower traits and self-compatibility

**Figure 2:**
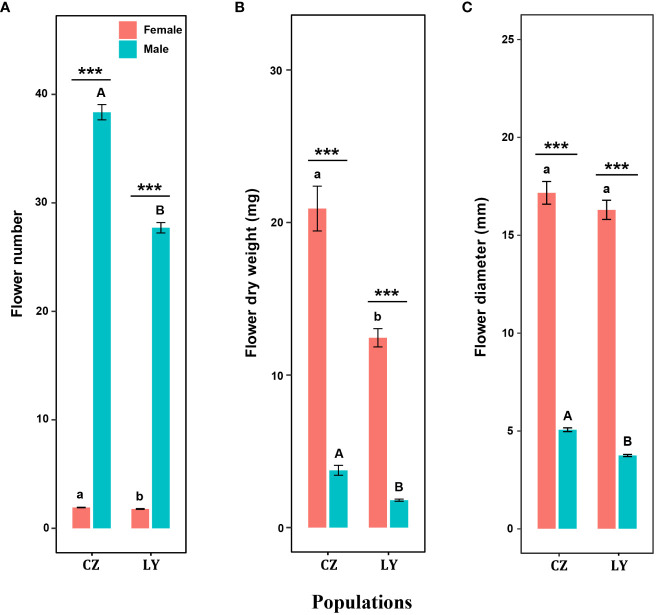
Effects of sex type on flower number **(A)**, flower dry biomass **(B)**, and flower diameter **(C)** in CZ and LY populations of *Akebia trifoliata*. Female flowers are indicated in red and male flowers are indicated in green. The heights of the columns and the error bars indicate the mean and standard error, respectively. Asterisks indicate significant differences between sex types in the same population (***P <0.001); different lowercase letters indicate significant differences between different populations for a given female flower trait, and uppercase letters indicate significant differences between different populations for male flower traits.

Flower number, flower dry biomass and flower sepal diameter were significantly influenced by sex and population size, and the interaction of these factors significantly influenced flower number and flower dry biomass (**Table 1**). For each inflorescence, the number of male flowers was significantly greater than that of female flowers in both CZ and LY populations (**Figure 2A**), but the per-flower dry biomass and diameter of female flowers were significantly larger than those of male flowers (**Figures 2B, C**). These results indicate that the inflorescence of *A*. *trifoliata* produced smaller male flowers (**Figure 2A**) and fewer larger female flowers (**Figures 2B, C**) in both the CZ and LY populations. Moreover, the number of flowers per plant, flower dry biomass, and flower diameter were higher in the CZ population than in the LY population (**Figure 2**).

**Table 1:** Results of the generalized linear model (GLM) testing whether the variation in flower number, flower size, and flower dry biomass was significantly influenced by sex type, population, and their interaction in *Akebia trifoliata*.

Sources	*DF*	Flower number		Flower size		Flower dry biomass	
		*χ^2^*	*P-*value	*χ^2^*	*P-*value	*χ^2^*	*P-*value
Sex type	1	6788.60	**<0.001**	763.38	**<0.001**	51.67	**<0.001**
Population	1	289.57	**<0.001**	13.33	**<0.001**	16.55	**<0.001**
Sex type × Population	1	9.85	**<0.001**	0.29	0.590	4.66	**0.031**

Significant results (P <0.05) are shown in bold.

**Figure 3:**
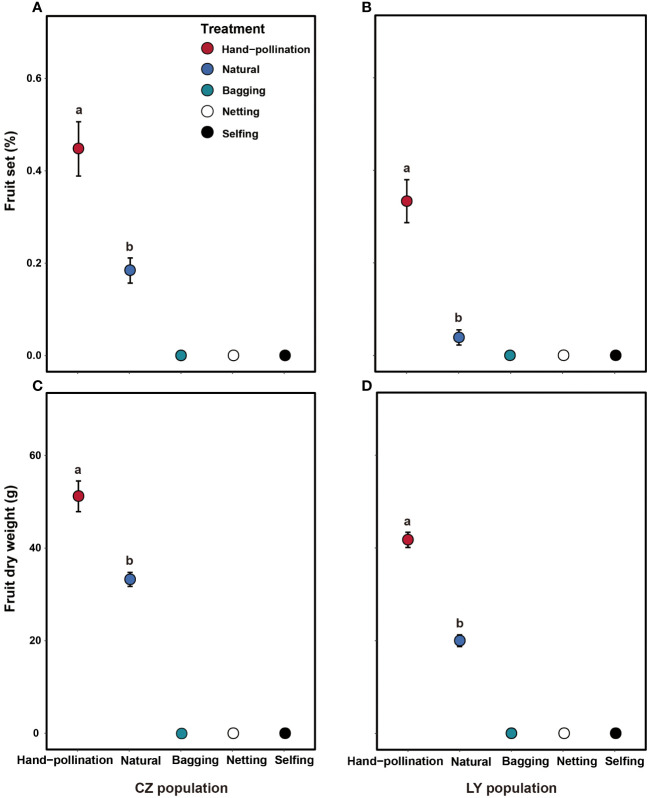
Effects of pollination treatment on fruit set **(A, B)** and dry fruit biomass **(C, D)** in the CZ (left) and LY (right) populations of *Akebia trifoliata*. Different pollination types are indicated by filled and colored dots. The heights of the error bars indicate standard errors. Different lowercase letters indicate significant differences among the treatments.

Bagged flowers without manipulation in the two populations did not set any fruits (**Figures 3A, B**), indicating that no apomixis occurred. Netted flowers in the two populations also did not set any fruit (**Figures 3A, B**); therefore, wind pollination is unlikely and fruiting likely requires pollinators. The self-pollinated flowers in the two populations did not produce any fruit (**Figures 3A, B**), indicating that *A*. *trifoliata* is self-incompatible.

### 3.2 Fruit production and pollinator probing preference

**Figure 4:**
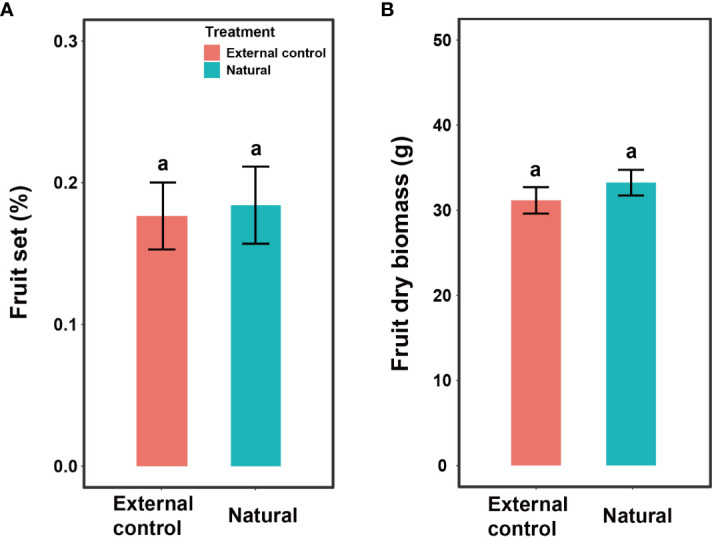
Effects of pollination treatment on fruit set per flower **(A)** and dry fruit biomass **(B)** of *Akebia trifoliata* in the CZ population. The natural group indicates that the inflorescence was pollinated by hand, and the external control group indicated that the inflorescence was intact. The heights of the error bars indicate mean and standard errors. Different lowercase letters indicate significant differences among pollination treatments.

Fruit set and fruit dry biomass were significantly influenced by pollination treatment, population, and their interactions (**Table 2**). Compared to natural pollination, hand pollination significantly enhanced fruit set and fruit dry weight in the CZ population (**Figures 3A, C**). Similarly, fruit set and dry weight were also significantly enhanced by hand pollination in the LY population (**Figures 3B, D**). The PL index for fruit set was 0.192 for the CZ cultivated population and 0.783 for the LY wild population, i.e., pollen supplementation increased the fruit set by 23.8% and 360%, respectively, indicating that the monoecious species *A*. *trifoliata* experienced severe pollen limitation and that pollinator services were necessary during the reproductive process. In addition, neither fruit set nor fruit weight differed significantly between the natural pollination and external control treatments in the CZ-cultivated population (**Figure 4**), but supplemental pollination resulted in significantly higher fruit set and fruit weight than in both the natural pollination and external control treatments (**Figures 3A, B**, **4**). Therefore, there was no evidence of resource reallocation among the flowers.

**Table 2:** Results of the generalized linear model (GLM) testing whether the variation in fruit set and fruit dry weight was significantly influenced by different pollination treatments, populations, and their interaction in *Akebia trifoliata*.

Sources	*DF*	Fruit set		*DF*	Fruit dry weight	
		*χ^2^*	*P-*value		*χ^2^*	*P-*value
Treatment	4	683.296	**<0.001**	1	28.865	**<0.001**
Population	1	27.061	**<0.001**	1	13.658	**<0.001**
Treatment × Population	4	19.044	**<0.001**	1	0.339	0.561

Significant results (P <0.05) are shown in bold.

**Figure 5:**
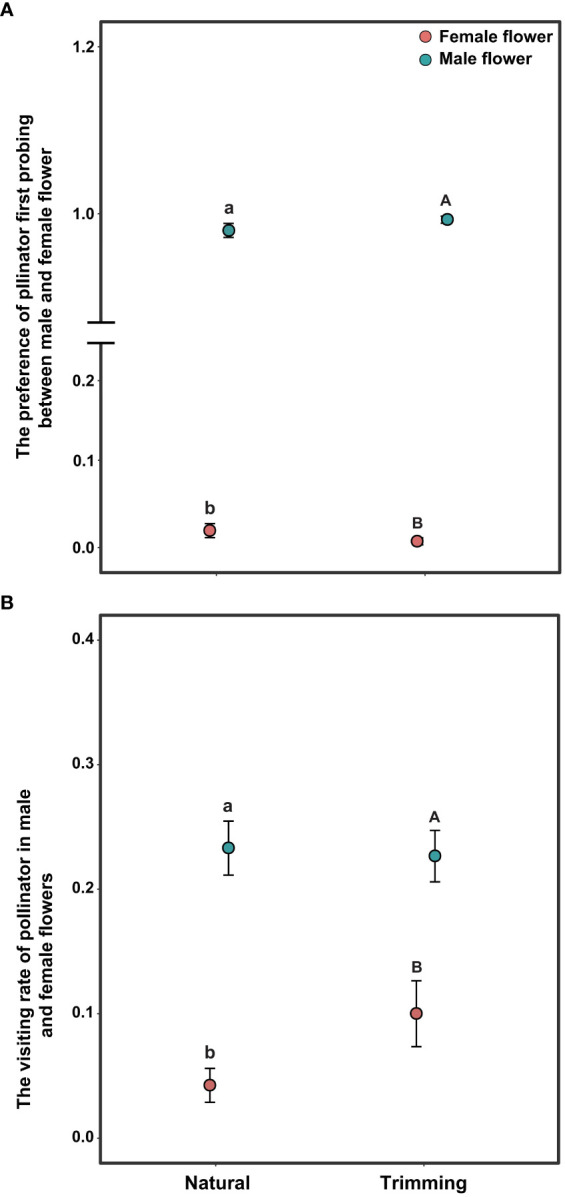
Differences in pollinator behavior on the first probing preference **(A)** and per flower visitation rate **(B)** of *Akebia trifoliata* under natural and trimming conditions. Different pollination types are indicated by filled and colored dots. The heights of the dots and error bars indicate mean and standard error, respectively. Different lowercase letters indicate significant differences within the natural treatment; and different uppercase letters indicate significant differences within the trimming treatment.

The pollen-collecting honeybee is the only pollinator of *A*. *trifoliata*. In 2019, sex significantly influenced the pollinator’s first probing preference and visitation rate per flower within the inflorescence (**Table 3**), indicating that honeybees exhibited a significant male flower probing bias (**Figure 5A**), and the average visitation rate of male flowers was significantly higher than that of female flowers (**Figure 5B**). In addition, the effects of trimming and its interaction with sex were not significant for the two variables (**Table 3**). The pollinator also exhibited male flower probing bias and a higher visitation rate on male flowers (**Figure 5**).

**Table 3:** The results of generalized linear and mixed models (GLM and GLMM) testing whether the variation in pollinator first probing preference and per flower visiting rate was significantly influenced by sex type, trimming treatment, and their interaction in *Akebia trifoliata*.

Sources	*DF*	Probing bias		Visiting rate	
		*χ^2^*	*P-*value	*χ^2^*	*P-*value
Sex type	1	20.776	**<0.001**	58.591	**<0.001**
Trimming	1	0.038	0.845	0.660	0.417
Sex type × Trimming	1	1.756	0.183	0.661	0.416

Significant results (P <0.05) are shown in bold.

### 3.3 Self-interference

**Figure 6:**
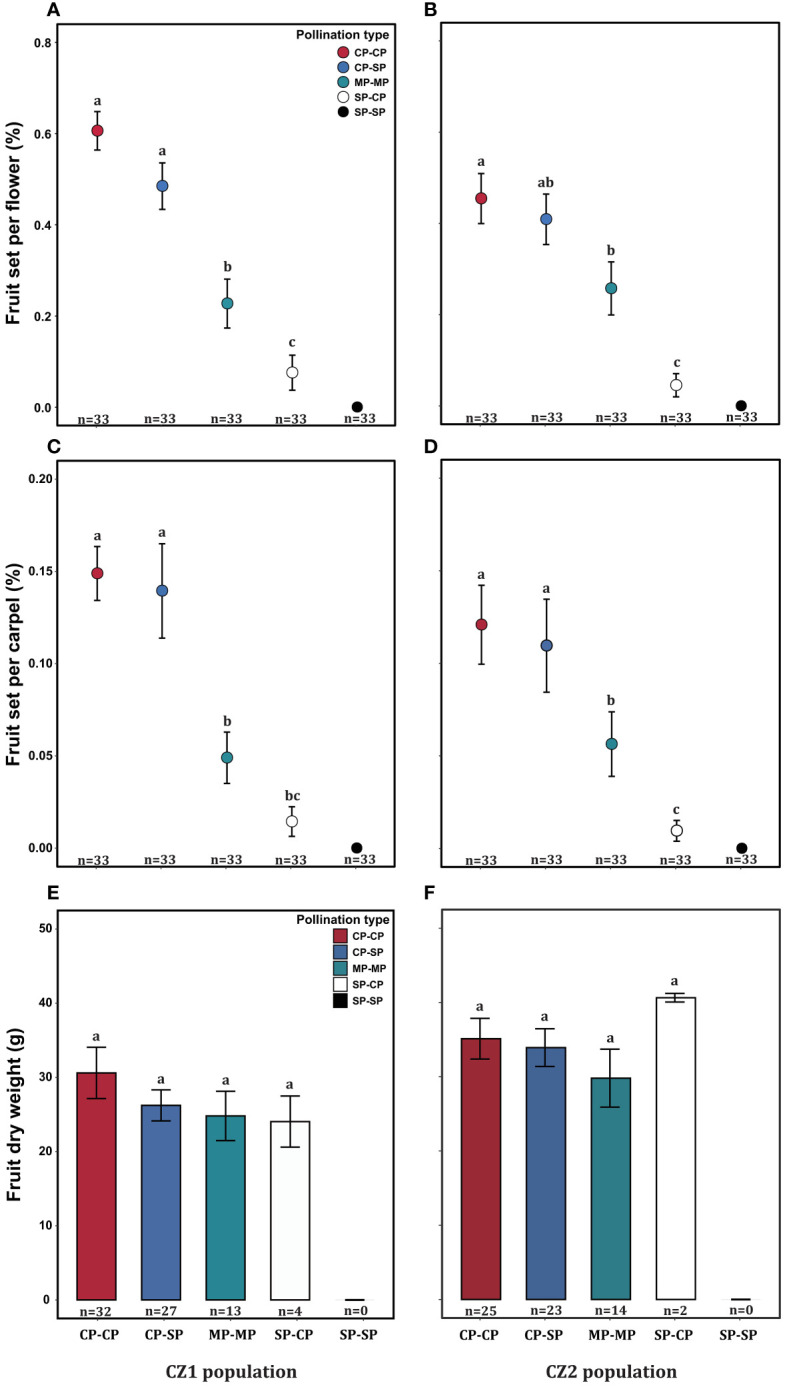
Effect of pollination type on fruit set per flower **(A, B)**, fruit set per carpel **(C, D)**, and fruit dry biomass **(E, F)** of *Akebia trifoliata* in the CZ1 (left) and CZ2 (right) populations. The heights of the error bars indicate the means and standard errors. Different lowercase letters indicate significant differences among the pollination treatments.

The fruit set per flower or carpel varied significantly among treatments but was not influenced by population or their interaction (**Table 4**). The dry biomass per fruit was significantly influenced by population, but not by treatment and their interaction (**Table 4**). In each population, the self-pollinated flowers did not set any fruit, further confirming that *A*. *trifoliata* was self-incompatible (**Figure 6**). Compared with pure cross-pollination, self-pollination after cross-pollination did not significantly affect fruit set (**Figures 6A-D**), but simultaneous self- and cross-pollination significantly reduced fruit set (**Figures 6A-D**). In addition, self-pollination prior to cross-pollination yielded a significantly lower fruit set than simultaneous self- and cross-pollination (**Figures 6A-D**). However, the dry biomass per fruit was not significantly different, regardless of pollination treatment (**Figures 6E, F**).

**Table 4:** Results of the generalized linear and mixed models (GLM and GLMM) testing whether the variation in fruit set per flower or carpel (a) and fruit dry biomass (b) were significantly influenced by different pollination treatments, populations, and their interaction in *Akebia trifoliata*.

Sources	*DF*	Fruit set per flower (%)		*DF*	Fruit set per carpel (%)		*DF*	Dry biomass (g)	
		*χ^2^*	*P-*value		*χ^2^*	*P-*value		*χ^2^*	*P-*value
Treatment	4	288.766	**<0.001**	4	267.881	**<0.001**	3	2.534	0.458
Population	1	2.159	0.125	1	1.542	0.250	1	6.530	**0.011**
Treatment × Population	4	2.257	0.649	4	1.232	0.900	3	1.068	0.785

Significant results (P <0.05) are shown in bold.

**Figure 7:**
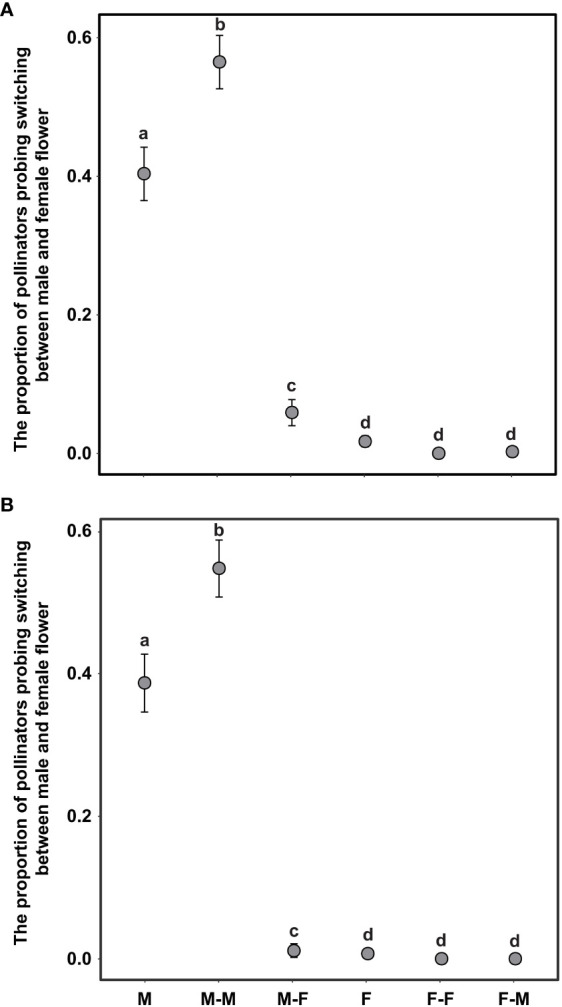
Difference in pollinator probing switching mode in the natural **(A)** and trimming **(B)** treatments in *Akebia trifoliata*. M and F indicate that the pollinator probed only male and female flowers, respectively; MM and FF indicate the switching of probing within male and female flowers, respectively; MF indicates that the pollinator probed male flowers followed by female flowers, and FM was the opposite of MF. The heights of the dots and error bars indicate the means and standard errors. Different lowercase letters indicate significant differences between natural and trimming treatments.

The switching modes of pollinator probing within inflorescences can significantly influence the self-interference. In the present study, the different switching modes of pollinator probing within inflorescences differed significantly in both the natural and trimming treatments (*df* = 5, *χ* ^2 =^ 1893.14, *p <*0.001 and *df* = 5, *χ* ^2 =^ 1,282.42, *p <*0.001 in the natural and trimming treatments, respectively). In the natural treatments, 40.0% of honeybees visited only male flowers, 55.0% of honeybees switched between male flowers, and only 1.3% of honeybees visited female flowers (**Figure 7A**). Similar switching modes of pollinator probing were observed in the trimming treatments (38.8% of honeybees visited only male flowers and 55.0% of honeybees switched between male flowers), and female flowers were rarely visited by pollinators (**Figure 7B**). Additionally, the trimming treatment did not influence the M, F, MM or FF switching modes (**Table 5**), but the MF and FM switching modes were significantly influenced (**Table 5**).

**Table 5:** The results of the generalized linear model (GLM) testing whether the pollinator probing switching modes were significantly influenced by the trimming treatment of *Akebia trifoliata*.

Sources	*DF*	*χ^2^*	*P-*value
Male	1	0.273	0.550
Female	1	1.252	0.495
Male-Male	1	1.349	0.180
Male-Female	1	28.082	**<0.001**
Female-Male	1	29.286	**0.043**
Female-Female	–	–	–

The female−female switching mode could not be detected; hence, it was represented by an em-dash. Significant results (P <0.05) are shown in bold.

## 4 Discussion

We successfully evaluated self-compatibility and self-interference in the monoecious species *A*. *trifoliata* and directly explored the effect of pollinator probing preference and switching mode between female and male flowers on self-interference. Our results revealed that lower levels of pollinator services reduced reproductive fitness in this self-incompatible species, indicating that artificial cross-pollination and release of pollinators are necessary to increase the fruit yield of this economically important species. In addition, self-interference occurred in *A*. *trifoliata*, but the male flower probing bias and switching mode of pollinators were inconsistent with our assumption, potentially reinforcing the degree of self-interference. Below, we discuss these results and their interpretations in greater depth as well as their implications for the evolution and maintenance of sexual systems in *A*. *trifoliata*.

### 4.1 Self-compatibility and pollen limitation reduced fruit production

The flower traits of *A*. *trifoliata* suggest that there is possible wind pollination in *Akebia* species because of characteristics such as unisexual flowers, absence of nectaries, inflorescence shape, dry powdery appearance of pollen grains (**Figure 1**), and immediate abscission of staminate flowers after anthesis (Li et al., 2010). However, the netted flowers in our pollination experiments did not set any fruit in either the cultivated or wild populations of *A*. *trifoliata* (**Figure 2**), demonstrating that wind pollination is unlikely for *A*. *trifoliata*. Similarly, Kawagoe and Suzuki (Kawagoe and Suzuki, 2002; Kawagoe and Suzuki, 2005) did not demonstrate a role of wind in the pollination of *A. quinata*. Moreover, geitonogamous self-pollination treatment did not result in any fruit production, indicating that *A*. *trifoliata* is completely self-incompatible (Zapata and Arroyo, 1978). These results demonstrate that *A*. *trifoliata* is a self-incompatible monoecious species, which is consistent with the findings in *A. quinata* (Kawagoe and Suzuki, 2002; Kawagoe and Suzuki, 2005). In addition, *A*. *trifoliata* has a strong male-biased floral sex ratio, and few female flowers open for more than half a month. Both characteristics are favorable for successful pollination. However, hand pollination significantly increased fruit production in both cultivated and wild populations, suggesting that *A*. *trifoliata* experiences pollen limitation under natural conditions (**Figure 3**). The male flower probing bias of pollinators was responsible for pollen limitation and reduced fruit production (**Figures 3**, **5**).

Pollinator-mediated decreases in pollen quantity and quality are the primary reasons for pollen limitation and fruit production, particularly in self-incompatible and animal-pollinated species (Burd, 1994; Larson and Barrett, 2000; Ashman et al., 2004; Knight et al., 2005). In a survey of 482 studies, Knight et al. (2005) concluded that 284 (63%) species exhibited severe pollen limitation in fruit production at some sites or during some years (the index of pollen limitation was 0.52). In our study, the average pollen limitation index for fruit set in *A*. *trifoliata* was 0.488, which is very close to that reported in a previous study (Knight et al., 2005), indicating that *A*. *trifoliata* also experienced severe pollen limitation in fruit production. These results are understandable because the only pollinator of *A*. *trifoliata* was the honeybee, and the visitation rate was quite low; the visitation rate of female flowers was lower than that of male flowers (**Figure 5**). Thus, pollinator probing behavior could be the primary reason for low fruit production in *A*. *trifoliata*. A low pollinator visitation rate and pollen limitation in fruit production have also been observed in *A. quinata* by Kawagoe and Suzuki (2002). The difference is that pollen limitation was not reported directly in their study, and our study not only demonstrated the existence of pollen limitation, but also measured it successfully (**Figure 3**).

In pollen limitation studies, if resources are diverted to supplementally pollinated flowers or inflorescences, pollen supplementation at the flower or inflorescence level may lead to a significantly higher estimate of pollen limitation than manipulations performed at the whole-plant level (Zimmerman and Pyke, 1988; Knight et al., 2006). Whole-plant manipulations are often regarded as more accurate because resources cannot be differentially reallocated among fruits based on pollen quantity or quality. However, we could not submit whole *A*. *trifoliata* plants to control or experimental treatments because an individual *A*. *trifoliata* plant can produce hundreds of inflorescences. Therefore, following previous studies (Wesselingh, 2007; Fernández et al., 2012; Dai et al., 2018), we used two complementary inflorescence controls: one from the treated plants and the other from the untreated plants. We found that supplementally pollinated inflorescences had higher fruit set and fruit weight than naturally pollinated inflorescences from both manipulated and non-manipulated plants (**Figure 3**). However, naturally pollinated inflorescences from manipulated and non-manipulated plants did not differ in fruit set or fruit weight (**Figure 4**), indicating that pollen added to some inflorescences did not lead to resource diversion from naturally pollinated inflorescences on the same plants. Furthermore, because the fruits came from the same position within the plants (including basal, middle, and distal), the flower position did not significantly influence fruit set at our study site. In summary, the magnitude of pollen limitation is unlikely to have been overestimated in our study, and the lower fruit production in the present study was real; therefore, artificial cross-pollination and the release of pollinators are necessary to increase the fruit yield of this economically important species under natural conditions.

### 4.2 Pollinator probing preference and self-interference are beneficial for the evolution and maintenance of sexual systems

The production of hundreds of inflorescences and the large overlap of female and male phases within an inflorescence highlight the importance of assessing self-interference in *A*. *trifoliata*. In the present study, fruit set and fruit weight were similar between cross-pollination and cross-pollination, followed by self-pollination (**Figure 6**). However, in comparison with cross-pollination, simultaneous self- and cross-pollination significantly reduced fruit set and fruit weight in both the CZ and LY populations (**Figure 6**), and self-pollination prior to cross-pollination had the most significant effect on reproductive output (**Figure 6**), leading to almost complete reproductive failure in the related species *A. quinata*. In *A. quinata*, prior self-pollination and simultaneous self- and cross-pollination yielded similar numbers of fruits, but both resulted in a lower fruit set than that of cross-pollination (Kawagoe and Suzuki, 2005). These results demonstrate that self-interference occurs and successfully reduces reproductive fitness in *A*. *trifoliata*, which is more susceptible to self-interference than *A. quinata*, indicating that the effects of self-interference on fruit and seed production vary depending on the species (Barrett, 2002a; Cesaro et al., 2004; Torres and Puntieri, 2013). However, the specific mechanisms underlying these effects remain unknown.

Regarding the source of self-interference in monoecious plants, probing preference and switching modes of pollinators are very important, especially in self-incompatible species. We hypothesized that the pollinator probing preference for female flowers can successfully reduce selfing and self-interference because female flowers are significantly larger than male flowers, which can enhance attractiveness to pollinators. However, pollinators exhibited a significant male flower preference in *A*. *trifoliata* (**Figure 5**), which was contrary to our expectations. First, we hypothesized that the clustered male flowers had a significantly higher advertising effect on pollinators than female flowers (the male flowers were 19 times as numerous as the females in the CZ population, and 14 times in the LY population); thus, from the perspective of the pollinator, a larger male flower can successfully enhance attractiveness within inflorescences compared to smaller female flowers. Moreover, the lower visitation frequency of female flowers may be related to the pollinator probing path. In the present study, we observed the acropetal foraging behavior of honeybees within the inflorescences of *A. trifoliata*. This behavior is consistent with previous results (Zhang et al., 2006), indicating that female flowers were not preferentially visited by pollinators. Furthermore, the male flowers also provided the only reward pollen to honeybees, and the female flowers did not provide any reward to pollinators (lacking both pollen and nectar). These results imply that flower number and pollen are more important than flower size in enhancing the attractiveness of pollinators to nectarless plants of *A. trifoliata*. Second, most honeybees probed only male flowers or probed male flowers preferentially (**Figure 7**), and very few honeybees probed female flowers or switched between female flowers (**Figure 7**). These switching modes of pollinator probing within inflorescences could reinforce self-interference because most pollinators preferentially probe male flowers and spread selfing pollen to female flowers within inflorescences, resulting in self-interference in self-incompatible *A*. *trifoliata*. In addition, although the lower visitation frequency of pollinators to female flowers could theoretically reduce self-interference within inflorescences, we rarely observed female flower probing preference and switching from female flowers to male flowers under natural conditions (**Figures 5**, **7**). In summary, *A*. *trifoliata* cannot avoid self-interference under the mediation of male-biased pollinator probing and switching modes under natural conditions. However, the mechanism by which monoecious species effectively avoid self-interference requires further experimental studies. Although the mechanism underlying self-interference avoidance remains unknown, it is clear that pollinator behavior causes strong geitonogamy, resulting in pollen discounting, stigmatic clogging, self-pollination prior to cross-pollination, and reduced fruit production in *A*. *trifoliata* (**Figures 6**, **7**).

The present study and two previous studies have shown that geitonogamous self-pollination could significantly reduce out-crossed fruit and/or seed production in self-incompatible monoecious plants (Kawagoe and Suzuki, 2005; Torres and Puntieri, 2013). Selfing can promote the evolution of plant sexual systems, particularly the evolution of separate sexes from combined sexes (Charlesworth and Charlesworth, 1978; Lloyd, 1982; Charlesworth, 1999). The theory of the evolution of dioecy from monoecy assumes that selfing combined with inbreeding depression in co-sexual populations favors individuals with reduced allocation to male sex function, thereby reducing selfing rates (Charlesworth and Charlesworth, 1978). Therefore, significant selfing in monoecious populations can provide evidence that inbreeding avoidance is involved in evolutionary transitions from monoecy to dioecy (Renner and Won, 2001; Dorken et al., 2002; Dorken and Barrett, 2003; Kawagoe and Suzuki, 2005). Coincidentally, the results of the present study indicate that *A*. *trifoliata* is more susceptible to self-interference and can successfully avoid selfing because it is a self-incompatible species. This evidence provides the potential dynamics for the evolution of monoecy. In other words, monoecy may be a way to reduce self-interference by separating male and female functions between flowers; however, this speculation needs to be supported by further experimental results.

In conclusion, we demonstrated that pollinators probing male flowers preferentially cause significant self-interference and reinforce pollen limitation on reproduction in the new high-value crop *A*. *trifoliata*. Therefore, artificial supplementary pollination is necessary to increase the fruit yield. Moreover, our results did not support the hypothesis that the mechanism of monoecious species can effectively avoid self-interference, as precicted by theory. Exploring the role of pollinator services during reproduction in monoecious species will help us better understand the mechanisms of self-interference avoidance in future studies.

## Data availability statement

The original contributions presented in the study are included in the article/**Supplementary Material**. Further inquiries can be directed to the corresponding authors.

## Author contributions

B-XW and G-XC conceived the study. G-XC designed it. B-XW and NX performed the experiments, with NX helping to conduct them. HW and G-XC collected and analyzed the data and wrote the manuscript. All authors contributed to the article and approved the submitted version.
